# Resveratrol and ω-3 PUFAs Promote Human Macrophage Differentiation and Function

**DOI:** 10.3390/biomedicines10071524

**Published:** 2022-06-28

**Authors:** Joseph Schwager, Albine Bompard, Daniel Raederstorff, Hubert Hug, Igor Bendik

**Affiliations:** 1 DSM, HNC, Innovation, Global R&D Center, Wurmisweg 567, CH-4303 Kaiseraugst, Switzerland; daniel.raederstorf@orange.fr (D.R.); hubert.hug@dsm.com (H.H.); igor.bendik@dsm.com (I.B.); 2 DSM, HNB, BDT, Toxicology & Kinetics, Wurmisweg 567, CH-4303 Kaiseraugst, Switzerland; albine.bompard@dsm.com

**Keywords:** innate immune response, macrophage differentiation, alternative activated macrophages, pro-inflammatory macrophages, nutrients, resveratrol, ω-3 polyunsaturated fatty aids (ω-3 PUFAs), chemokines, cytokines

## Abstract

Monocytes differentiate into M1 and M2 macrophages, which are classically activated by microbial products such as LPS or IFN-γ and interleukins (e.g., the anti-inflammatory and T_h_2 promoting IL-4), respectively. The contribution of nutrients or nutrient-based substances such as ω-3 polyunsaturated fatty acids (ω-3 PUFAs) and resveratrol (Res) on the differentiation and function of M1 and M2 macrophages was evaluated. THP-1 cells and peripheral blood mononuclear cells (PBMCs) were differentiated into M1 and M2 cells and activated with LPS/IFN-γ or IL-4/IL-13. Macrophage lineage specific surface determinants (e.g., CD11b, CD11c, CD14, CD206, CD209, CD274, HLA-DR, CCR7, CCR2) were analysed by cytofluorometry. Res and ω-3 PUFAs altered CD14, CD206, CD274 and HL-DR surface expression patterns in M1 and M2 macrophages differentiated from PBMC. LPS/IFN-γ or IL-14/IL-13 activated macrophages subpopulations, which secreted cytokines and chemokines as measured by multiplex ELISA. Res and ω-3 PUFA reduced IL-1β, IL-6, TNF-α, CXCL10/IP-10, CCL13/MCP-4 and CCL20/MIP-3α in LPS/IFN-γ activated human leukaemia THP-1 cells, which is indicative of a dampening effect on M1 macrophages. However, Res increased M1 prototypic cytokines such as IL-1β or IL-6 in macrophages derived from PBMCs and also modified the expression of IL-12p70. Collectively, Res and ω-3 PUFAs distinctly promoted the differentiation and function of M1 and M2 macrophages. We conclude that these substances strengthen the macrophage-mediated effects on the innate and adaptive immune response.

## 1. Introduction

Macrophages have numerous functions in the innate and adaptive immune response and play a key role in tissue homeostasis (e.g., reviewed in [1,2]). They are derived from bone marrow myeloid progenitor cells and differentiate from monocyte precursor cells into macrophages subtypes. Their differentiation pathways are influenced and determined by factors from their microenvironment. Colony-stimulating factors (CSF) such as GM-CSF and M-CSF induce distinct macrophages populations, termed M1 and M2, respectively, which exhibit phenotypic heterogeneity and plasticity [3,4,5,6,7] and can be generated in vitro [8,9,10]. M1 macrophages are pro-inflammatory and are activated by toll-like receptor (TLR) ligands or/and LPS/IFN-γ [7], whereas M2 macrophages are stimulated by IL-4/IL-13 and have anti-inflammatory and immune-suppressive functions [2,11,12,13,14]. The different macrophage subtypes have genuine phenotypes and surface determinants and express specific sets of marker genes [7,9,15,16,17,18] (see Table 1 for phenotypical and functional markers). For instance, CD14, CD274 are prototypic for M1 macrophages, while (increased) surface expression of MHC II [HLA-DR], CD204, CD206 and CD209 are hallmarks of M2 macrophages [2]. In the past, monocyte cell lines have been a valuable tool to study the functions of macrophages [19,20,21,22,23] or dendritic cells in vitro [24], since appropriate conditions for in vitro differentiation of macrophage (sub)-populations have shown to be difficult.

Colony stimulating factors mainly determine the fate of macrophage differentiation in conjunction with metabolic factors. In addition, nutrients and nutritional substances modulate cells of the adaptive and innate immune system including macrophages. Their anti-inflammatory and immunomodulatory effects could therefore contribute to skewing the balance between the M1/M2 phenotypes and functions. For instance, ω-3 polyunsaturated fatty acids (ω-3 PUFAs) have been shown to alter the functions of macrophages [28,29,30,31,32] and liver macrophages, i.e., Kupffer cells [33]. EPA and DHA reduced expression of pro-inflammatory cytokines and chemokines [30,31]. DHA favoured the differentiation of M2 phenotypes [34,35] and of RAW264.7 cells, a murine macrophage cell line [36,37,38,39]. Similarly, ω-3 PUFAs are associated with a shift in M1/M2 macrophage transition of Kupffer cells [33]. We and others have shown the anti-inflammatory effects of resveratrol (Res) on blood cells, macrophage/monocyte and endothelial cell lines after induction of the inflammatory response [40,41]. Res also modulated the M1/M2 balance in Kupffer cells [42].

The present study aimed evaluating the effects of nutrients on macrophage phenotypes and functions. For this purpose, peripheral blood mononuclear cells (PBMC) were differentiated into M1 and M2 cells and incubated with specific activators of M1 and M2. Similarly, the human leukaemia monocytic cell line THP-1, was treated with M1 and M2 activators. Phenotypes and functions of different cell populations were determined and related to M1 or M2 prototypic parameters. The results showed that Res and ω-3 PUFAs influenced the phenotypes and functions of M1 and M2 macrophages.

## 2. Materials and Methods

### 2.1. Reagents and Media

Ethanol, DMSO, phorbol 12-myristate 13-acetate (PMA), eicosapentaenoic acid (EPA), docosahexaenoic acid (DHA), resveratrol (Res), *E. coli* lipopolysaccharide (LPS, serotype 055:B5) and foetal bovine serum (FBS) were from Sigma (Sigma-Aldrich, Buchs, Switzerland). ω-3 PUFAs consisted of EPA and DHA, which were mixed at a 1:1 molar ratio. Cell culture reagents (RPMI 1640, DMEM, 2-mercaptoethanol and non-essential amino acids (NEAA)) were from Invitrogen (Life Technologies Europe B.V., Zug, Switzerland). Human recombinant IFN-γ, IL-4 and IL-13 were from Peprotech EC (London, UK). Monocyte attachment medium, macrophage detachment solution, M1 macrophages and M2 macrophage generation medium were from PromoCell (Heidelberg, Germany) and used according to the manufacturer’s instruction.

### 2.2. Isolation and Culture of Monocytes from Human Peripheral Blood Mononuclear Cells (PBMC)

Human peripheral blood leukocytes (PBL) were isolated from freshly drawn blood from healthy individuals as described previously [43]. Briefly, PBMC were isolated from total blood by Ficoll-Isopaque centrifugation, washed twice in an excess of PBS/EDTA (2 mM) to remove platelets and resuspended in culture medium. The purity of PBMC was analysed using the FSC/SSC cytofluorometric plot and was >80%. The PBMC population contained >25% of monocytes as determined by FSC/SSC analysis. PBMC were seeded in 12-well culture plates for 4 h at 37°/5% CO_2_ in humidified atmosphere. Non-adherent cells were subsequently removed. Adherent cells were washed and cultured in complete RPMI 1640 medium (containing 10% FBS, 0.1 mM nonessential amino acids, 50 U/mL penicillin [P], 50 μg/mL streptomycin [S]) at conditions of M1 polarization [with GM-CSF], M2 polarization [with M-CSF] or without CSF [M0 polarization] for 6 days [7,18,44]. Where appropriate, substances were added to culture medium at the beginning of the polarization period. Macrophages were harvested after 6 days and processed for cytofluorometric analysis. Alternatively, culture media were renewed, and the cells activated with LPS (100 ng/mL)/IFN-γ (20 ng/mL) or interleukins (i.e., IL-4 and IL-13, 20 ng/mL of each interleukin). After 24 h, culture supernatants were recovered for chemokine and cytokine analysis. Cultured cells were detached and analysed by cytofluorometry. DNA contents and therefore the relative cell numbers obtained at different culture conditions were determined using the DNA intercalating dye (CyQUANT™)) according to the manufacturer’s instruction (Molecular Probes, www.thermofisher.com (accessed on 29 April 2022)).

### 2.3. THP-1 Cells

THP-1 cells, a human leukaemia monocytic cell line, were from Cell Lines Service (Eppelheim, Germany) and maintained at <2 × 10^5^ cells/mL in RPMI 1640 medium supplemented with P/S, 10% FCS and 2 mM L-glutamine. Cell adherence was induced by treatment with 320 nM phorbol 12-myristate 13-acetate (PMA). Substances were added 4–6 h after beginning of PMA treatment. Cells were then activated with LPS (100 ng/mL)/IFN-γ (20 ng/mL) and IL-4/IL-13 (20 ng/mL of each interleukin), respectively. Cell culture supernatants were recovered after 24 h and processed for chemokine and cytokine analysis. Where appropriate, THP-1 cells were detached, and the expression of surface determinant was measured by cytofluorometry. Cell numbers were determined with the CyQUANT™ DNA dye kit (Molecular Probes, www.thermofisher.com (accessed on 29 April 2022)).

### 2.4. Cytofluorometry

Cells were detached using the detachment medium (PromoCell, Heidelberg, Germany), washed with PBS, resuspended in HBSS/2% FCS/0.01% NaN_3_ (HFN), and incubated with fluorochrome-conjugated antibodies for 45 min at 4 °C. Cells were washed in HFN and resuspended in HFN containing 7-amino-actinomycin D (2.5 mg/mL). Fluorescence data of viable cells (2 × 10^4^ events) were acquired with a FACS Calibur™ cytofluorometer and evaluated with software provided by Becton and Dickinson (Mountain View, CA, USA). Appropriate isotype-specific controls were included in the cytofluorometric analysis. FITC-, PE- or APC-conjugated antibodies against CD11b, CD11c, CD14, CD-80, CD86, CD204, CD206, CD209, CD274, CCR7, CXCR2 and HLA-DR were obtained from eBioscience (Vienna, Austria), R&D Systems (Bio-Techne AG, Zug, Switzerland) and BD Pharmigen (San Diego, CA, USA) and used according to the manufacturer’s instructions.

### 2.5. Analysis of Cytokines, Chemokines and PGE_2_

Secreted analytes (cytokines, interleukins, chemokines, PGE_2_) were measured using multiparametric kits purchased from Bio-Rad Laboratories (Hercules, CA, USA) and EIA PGE_2_ assay kits (Cayman Chemicals, Ann Arbor, MI, USA). Data were acquired using the LiquiChip Workstation IS 200 (Qiagen, Hilden, Germany) and Spectramax (Molecular Devices, San Jose, CA, USA), respectively. Data evaluation was made with the LiquiChip Analyser software provided by Qiagen [45]. All data were normalized to the number of cells determined by Cyquant at the end of the experimental series.

### 2.6. Statistical Analysis

Data were evaluated using statistical tools described previously [46]. A *p* value < 0.05 (calculated with SPSS software from IBM SPSS v22, Dynelytics AG, Zürich, Switzerland by using Student’s *t* test or one-way ANOVA) was considered to reflect statistically significant differences.

### 2.7. Dosage Information

The final concentrations of substances in cell cultures were Res at 25 µM and omega-3 PUFAs at 20 µM. Unmodified Res had a plasma half-life of several hours [47], while omega-3 PUFAs persisted in plasma for days and weeks [48]. Dietary supplementation with Res and ω-3 PUFAs resulted in plasma concentrations of 2 µM [47] and 30 µM [48], respectively. Yet, transient cellular concentrations of substances might be considerably higher as shown for ascorbic acid (Schwager et al., 2015 [46] and references therein).

## 3. Results

### 3.1. Activation of THP-1 Cells with LPS/IFN-γ or IL-4/IL-13 Induced Phenotypic Changes That Are Modulated by Res and ω-3 PUFAs

**Figure 1:**
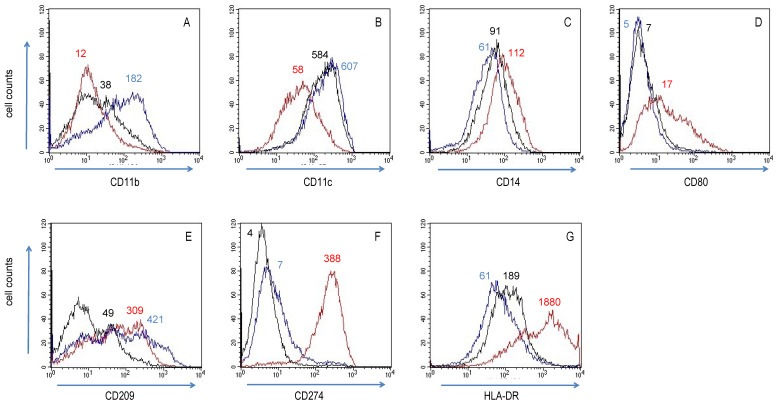
In vitro characterization of PMA-treated and activated THP-1 cells. Levels of (**A**) CD11b, (**B**) CD11c, (**C**) CD14, (**D**) CD80, (**E**) CD209, (**F**) CD274 and (**G**) HLA-DR expression were analysed by cytofluorometry in THP-1 cells, which had been cultured for 20 h in medium only (black), or activated with LPS/INF-γ (red) and IL-4/IL-13 (blue). Results are representative of two different experimental series, each carried out in duplicates. Mean fluorescence intensity (MFI) variations between curves are ≈2- to ≈50-fold due the logarithmic scale on the x-axis. MFI values are indicated for the major peak of the colour-coded cytofluorometric curves.

Initially, we examined the differentiation of a surrogate macrophage cell line and the effect of substances on markers of differentiation. To this goal, we treated THP-1 cells, a human leukaemia monocytic cell line, with PMA. This induced cell adherence and differentiation into macrophage-like cells. These mirrored the (operationally termed) M0 status of macrophages. PMA-treated cells were further incubated with LPS/IFN-γ and IL-4/IL-13, in order to induce M1 and M2 activation status, respectively [49]. Activated THP-1 cells underwent substantial phenotypic changes within 24 h. With regard to surface expression of macrophage polarization markers (see Table 1), M2 macrophage surface markers CD11b (integrin αM), CD11c (integrin αX), CD209 (DC-SIGN) and HLA-DR had a broad distribution on M0 (i.e., un-stimulated) THP-1 cells, while M1 macrophage surface markers CD14 (LPS receptor), CD80 (B7-1) and CD274 (B7-H1) determinants were more evenly expressed (Figure 1, see “Medium-only” profile). LPS/IFN-γ-treatment of THP-1 cells (to induce M1 activation status) resulted in increased surface density of CD14, CD80, CD274 and HLA-DR (Figure 1, “LPS/INF-γ” profiles); conversely, CD11b and CD11c density was lower on M1 THP-1 cells, whereas CD209 expression was unchanged. In comparison to M0 THP-1 cells, IL-4/IL-13-activated THP-1 cells (Figure 1, “IL-4/IL-13” profiles) expressed less CD14 and HLA-DR, but markedly more CD11b and CD209.

**Figure 2:**
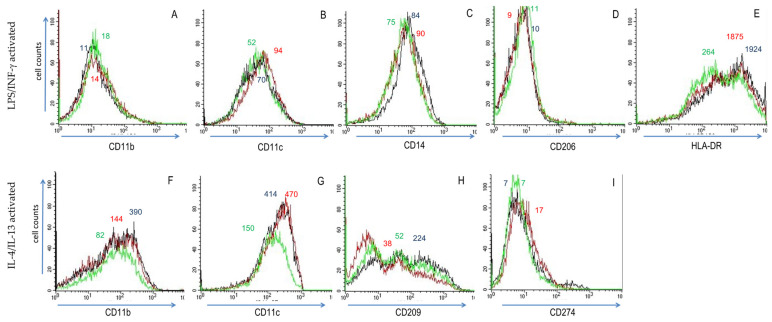
Effect of resveratrol (Res) and ω-3 polyunsaturated fatty acids (ω-3 PUFAs) on THP-1 cells activated with LPS/INF-γ (panels **A**–**E**) and IL-4/IL-13 (panels **F**–**I**). Levels of CD11b (**A**,**F**), CD11c (**B**,**G**), CD14 (**C**), CD206 (**D**), CD209 (**H**), CD274 (**I**) and HLA-DR (**E**) expression were analysed by cytofluorometry in THP-1 cells activated for 20 h in the absence of substances (black), with Res (red) and with ω-3 PUFAs (green). Substances induced MFI intensity differences of ≈2-fold (e.g., ω-3 PUFAs in CD11b (**A**)) to >10-fold (e.g., Res in CD209 [H]). MFI values are indicated for the major peak of the colour-coded cytofluorometric curves.

We observed subtle but significant changes in surface determinant expression when PMA-differentiated THP-1 cells were activated in the presence of Res or ω-3 PUFAs. These substances induced ~2 to ~100 fold variations in mean fluorescence levels. In LPS/IFN-γ -treated (i.e., M1) THP-1 cells, the substances had minor effects on CD11b surface expression, which was increased by Res and ω-3 PUFAs (Figure 2). Res and ω-3 PUFAs reduced CD14 surface expression, whereas CD11c was enhanced (Figure 2, panels A–E). Both Res and ω-3 PUFAs decreased CD14 and HLA-DR surface deposition. These investigations were extended to IL-4/IL-13-treated (i.e., M2) THP-1 cells. ω-3 PUFAs lessened levels of CD11b and CD11c (Figure 2F,G), but it had no significant impact on CD14 (not shown). While ω-3 PUFAs had only a minor effect on CD274 surface expression, Res augmented it, whereas it blunted CD209^high^ expression (Figure 2, panels F–I). Collectively, these substances modulated surface markers on THP-1 cells towards M2 phenotypes (revealed also by a reduced M1 surface marker expression).

Cells were also cultured in M1 and M2 differentiation medium, in order to measure possible effects of GM-CSF and M-CSF on THP-1 cells. No significant phenotypic changes were observed within 6 days of culture (data not shown).

### 3.2. Res and Omega-3 PUFAs Modulate M1 and M2 Differentiation of Macrophages Derived from Peripheral Blood Mononuclear Cells (PBMCs)

**Figure 3:**
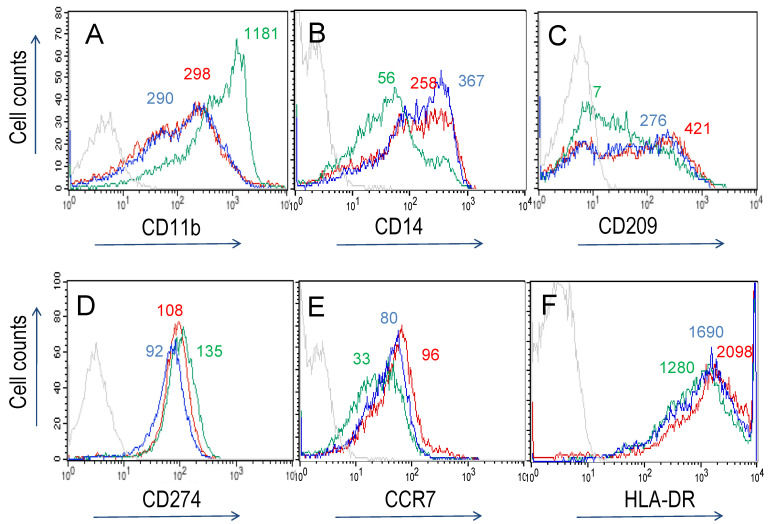
Phenotypic analysis of macrophages differentiated from adherent cells isolated from human peripheral blood and cultured for 6 days. M0, M1 and M2 macrophage phenotypes were obtained by culturing cells in basal medium, medium containing GM-CSF and M-CSF, respectively. Levels of CD11b (**A**), CD14 (**B**), CD209 (**C**), CD274 (**D**), CCR7 (**E**) and HLA-DR (**F**) expression in cells cultured in basal medium (red, M0 macrophages), GM-CSF (green, M1 macrophages) or M-CSF (blue, M2 macrophages) Substances induced MFI intensity differences of ≈2-fold (e.g., CD274 (**D**)) to >100-fold (e.g., CD14 (**B**)). Staining with isotype control antibodies: light grey. MFI values are indicated for the major peak of the colour-coded cytofluorometric curves (see also Supplementary Table S1). Only surface determinants where marked differences between cultured conditions were observed are shown.

Adherent cells, isolated from PBMCs, were cultured for 6 days with GM-CSF or M-CSF to induce M1 and M2 macrophages, respectively. They are also termed polarized M1 (M1p) and polarized M2 (M2p) macrophages [17,49,50]. At these culture conditions, M1 and M2 microphage differentiation is mirrored by the expression of distinct surface markers (Figure 3, Table 1). Cell-specificity of antibodies was determined by staining freshly isolated PBMCs (Figure S1). In vitro differentiated macrophages up-regulated CD14 and CD209, where the staining pattern indicated the appearance of intermediate-positive and bright-positive subpopulations (Figure 3). We compared surface expression on M0 macrophages (no CSF added to culture medium) to those of M1- or M2-differentiated macrophages. M1 macrophages displayed increased levels of CD11b and CD274, while the density of CD14 and CD209 was decreased (Figure 3, Table 1). M0 and M2-differentiated macrophages had similar surface density of CD11b, CD209 and HLA-DR (Figure 3). Collectively, the differences in expression of CD11b, CD14, and CCR7 (not shown) were diagnostic for M1 and M2 macrophage differentiation patterns (see also [7,18,44]. In addition, LPS/INF-γ activation on M1 and M2 macrophages caused down-regulation of CD14, CD11b, and CD11c (not shown).

**Figure 4:**
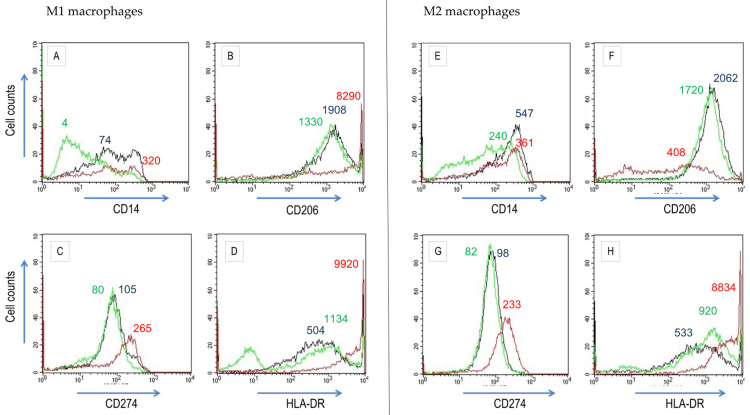
Res and ω-3 PUFAs alter the expression of surface determinants on M1 (GM-CSF) and M2 (M-CSF) macrophages differentiated from peripheral blood mononuclear cells. Adherent cells isolated from peripheral blood were cultured in medium containing GM-CSF to M1 macrophages (**A**–**D**) or M-CSF to M2 macrophages (**E**–**H**) with Res (red) or ω-3 PUFAs (green) or without substances (black) for 6 days. Levels of CD14 (**A**,**E**), CD206 (**B**,**F**), CD274 (**C**,**G**) and HLA-DR (**D**,**H**) were determined by cytofluorometry. MFI values are indicated for the major peak of the colour-coded cytofluorometric curves (see also Supplementary Table S2).

Next, cells were cultured in macrophage differentiation media containing Res or ω-3 PUFA. Cells, which differentiated in the presence of Res, formed aggregates (not shown). Res and ω-3 PUFAs altered the expression of some surface markers (Figure 4). During M1 macrophage differentiation, ω-3 PUFAs down-regulated CD14 surface deposition. On the contrary, Res significantly increased the expression of HLA-DR, CD206 (macrophage mannose receptor) and CD274. During M2 macrophage differentiation, Res markedly up-regulated CD274 and HLA-DR. ω-3 PUFAs also increased the HLA-DR cell population, diminished CD206 expression and augmented HLA-DR density. Taken together, these observations let us infer that Res and ω-3 PUFAs reduced M1 and promoted M2 macrophages populations.

In order to investigate whether the substances induced macrophage polarization, we cultured adherent PBMC during 7 days with Res or ω-3 PUFAs in M0 differentiation medium. The substances per se did not induce a distinct macrophage phenotype (not shown).

### 3.3. Res and Omega-3 PUFAs Influence the Functions of Activated THP-1 Cells

Following activation, M1 and M2 polarized macrophages secrete lineage-specific cytokines and chemokines as described in Table 1 [10]. In order to monitor similar changes in human leukaemia monocytic cell line, M1 and M2 status was induced in THP-1 cells by activation with LPS/INF-γ and IL-4/IL-13, respectively. Cells were cultured for 48 h and the secreted metabolites were determined. LPS/IFN-γ activated cells produced large amounts of cytokines and chemokines, while IL-4/IL-13 elicited the secretion of lower quantities of metabolites (Table 2). This indicates that THP-1 cells preferably responded to LPS/IFN-γ, which produced M1 prototype metabolites (e.g., TNF-α, IL-6 and CXCL10/IP/10; high M1/M2 ratio; Table 1) and less M2 prototypic metabolites (e.g., CCL13, CCL18, CCL20; low M1/M2 ratio).

**Figure 5:**
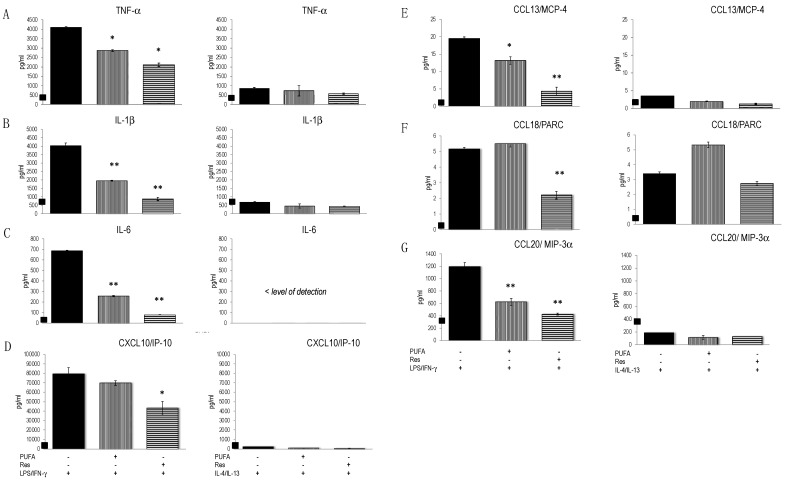
Secretion of cytokines and chemokines by THP-1 cells stimulated with LPS/IFN-γ or IL-4/IL-13. Cells were cultured for 20 h and culture supernatants analysed for interleukins/cytokines and chemokines. Black squares on the y-axis indicate the level of metabolite secreted by unstimulated cells. (**A**) TNF-α, (**B**) IL-1β, (**C**) IL-6, (**D**) CXCL10/IP-10, (**E**) CCL13/MCP-4, (**F**) CCL18/PARC, (**G**) CCL20/MIP-3α. Mean values ± standard deviation [pg/mL] of triplicates of a representative experiment (of three performed) are given. * *p* < 0.05, ** *p* < 0.01 (compared to LPS/INF-γ or IL-4/IL-13-activated cells).

Subsequently, we investigated the effects of substances on the functional properties of activate THP-1 cells. Res and ω-3 PUFAs mitigated the production of TNF-α, IL-1β, IL-6, CXCL10/IP-10, but also CCL13/MCP-4 and CCL20/MIP-3α. Res reduced cytokine secretion more potently than ω-3 PUFAs (Table 3, Figure 5). THP-1 cells activated with IL-4/IL-13 secreted similar amounts of cytokines and chemokines as unstimulated cells (except for CCL18/PARC), which indicates a low responsiveness of THP-1 cells to M2 activation signals. CCL18/PARC production was low at the cultured conditions used and only marginally altered by Res. Thus, Res and ω-3 PUFAs reduced the prototype M1 cytokines in THP-1 cells. Due to low basal expression after IL-4/IL-13 stimulation their effects on M2 polarized THP-1 cells could not be determined (Figure 5).

### 3.4. Res and ω-3 PUFAs Modulated the Secretion Pattern of PBMC-Derived M1 and M2 Macrophages

**Figure 6:**
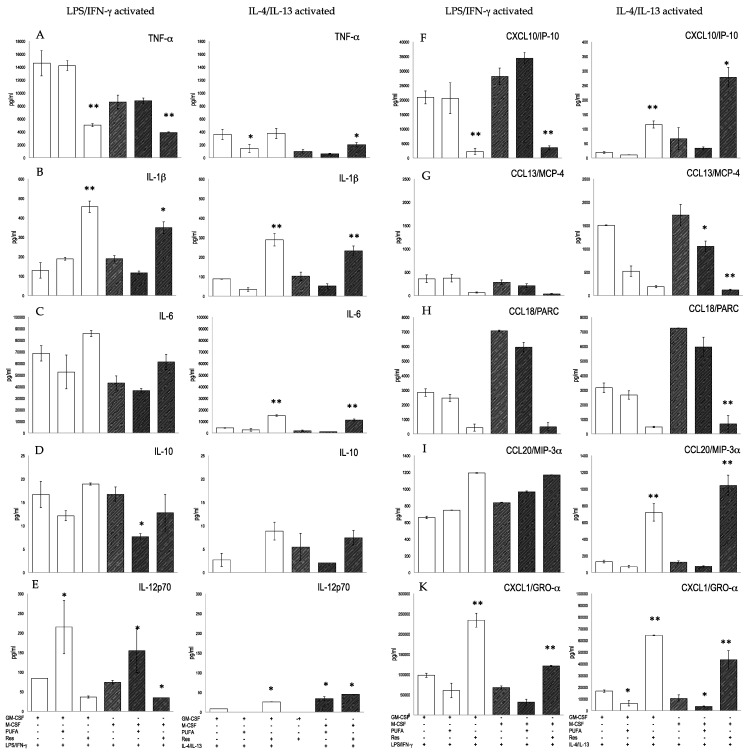
Secretion of cytokines and chemokines by macrophages differentiated from PBMCs. Adherent cells isolated from peripheral blood were cultured in GM-CSF (open bars) and M-CSF (hatched bars) for M1 and M2 differentiation, respectively, for 7 days, followed by activation with LPS/IFN-γ or IL-4/IL-13 for 24 h. (**A**) TNF-α (**B**) IL-1β, (**C**) IL-6, (**D**) IL-10, (**E**) IL-12p70, (**F**) CXCL10/IP-10, (**G**) CCL13/MCP-4, (**H**) CCL18/PARC, (**I**) CCL20/MIP-3α, (**K**) CXCL1/GRO-α. Mean values ± standard deviation [pg/mL] of triplicates of a representative experiment (of four performed) are given. * *p* < 0.05, ** *p* < 0.01 (vs. activated cells).

The secretion of prototype chemokines and cytokines was determined in different macrophage populations derived from PBMCs (Table 1). During a 7 days differentiation period, secreted chemokines and cytokines were similar at various culture conditions (Table 2, Figure 6). For instance, TNF-α and IL-6 levels were ≈900 pg/mL and ≈25 ng/mL at all conditions (Table 2). In addition, the M2 prototype chemokines CCL18/PARC and CXCL1/GRO-α were similar. This implies that during the in vitro polarization, PBMCs-derived M1 and M2 macrophages released similar amounts of M1 and M2 prototype chemokines and cytokines. This contrasts with different phenotypes observed at various differentiation procedures (Figure 3). Consequently, the pattern of secreted cytokines and chemokines did not fully match the phenotypes of in vitro polarized un-activated macrophages.

M1p macrophages responded to LPS/IFN-γ stimulation since levels of TNF-α were higher than in the un-stimulated counterparts (Figure 6). Similar observations were made for CXCL10/IP-10. M1p macrophages responded to IL-4/IL-13 activation with an excess of CCL13/MCP-4 and CCL18/PARC, which were significantly higher than in M1p medium-treated cells. M2p macrophages responded to IL-4/IL-13 activation with an increased secretion of CCL13/MCP4 and CCL18/PARC, whereas TNF-α, IL-6, CXCL10/IP10 and CCL20/MIP3α were secreted at lower amounts or barely matched the levels of un-stimulated cells. Yet, M2p macrophages still retained full responsiveness to LPS/IFN-γ, since they produced most of the metabolites at similar levels as LPS/INF-γ activated M1p macrophages Consequently, we observed only a quantitative functional difference between M0p, M1p and M2p macrophages. The LPS/IFN-γ to IL-4/IL-13 ratio, however, indicated that TNF-α or CXCL10/IP10 and CCL3/MCP-4 were functional markers for M1p and M2p macrophages respectively (Table 4). In contrast, PGE_2_ production was a specific read-out for M1p activated macrophages: LPS/IFN-γ induced significant levels of PGE_2_ only in M1p rather than in M0p and M2p cells (Figure S2).

Next, we investigated the impact of substances on the function of PBMCs-derived M1 and M2 macrophages. Both Res and ω-3 PUFAs modulated the expression of metabolites (Figure 6). ω-3 PUFAs only marginally influenced the cytokines and chemokines produced by PMBC derived M1 or M2 macrophages. Res enhanced the secretion of IL-1β, TNF-α and CCL20/MIP-3α, but it blunted CCL18/PARC production. We studied the impact of substances on the metabolites, that were secreted by activated M1p macrophages (Figure 6). The production of TNF-α and CXCL10/IP-10 was impeded by Res in M1p macrophages regardless of the activation signals (i.e., LPS/IFN-γ or IL-4/IL-13), whereas IL-1β production was increased in M1p macrophages. IL-6 was moderately blunted by ω-3 PUFAs in M1p and M2p populations. IL-12p70 was enhanced by ω-3 PUFAs and impeded by Res, whereas both substances had no significant impact on the expression of IL-10. We also observed significant effects of Res on IL-4/IL-13 activated M1 and M2 macrophages: the production of CXCL10/IP-10, CCL20/MIP-3α and CXCL/GRO-α was increased, while the M2 prototype chemokines CCL13/MCP-4 and CCL18/PARC were secreted at a lower amount by Res-treated cells.

## 4. Discussion

In the current study we evaluated whether nutritional substances contributed to the development and differentiation of macrophage populations and functions. In vitro polarization and activation procedures to induce M1 and M2 macrophages were applied to the human leukaemia monocytic cell line, THP1, and to cells, that differentiated from adherent human PBMCs. THP-1 cells might be considered to represent an M1 pre-differentiated macrophage line and therefore less inducible to differentiate into both M1 and M2 macrophages [49]. Accordingly, THP-1 cells did not further differentiate in the presence of colony stimulating factors including GM-CSF or M-CSF. However, they responded to M1 activation signals (i.e., LPS and INF-γ) and less to M2 activation signals (i.e., IL-4/IL-13). This suggests that THP-1 cells were biased to the M1 phenotype. Alternatively, the differentiation into M2 macrophages requires more complex interactions between chemokines and cytokines, which were not met in the THP-1 culture conditions procedure ([49]; this study). It should be noted that cell lines such as THP-1 cells were valid surrogates but not perfect counterparts of the PBMC derived macrophage populations [49].

Macrophages derived in vitro from adherent PBMCs differentiated into various macrophage populations. In two seminal studies, the features of M1 and M2 macrophage populations were identified at the level of gene transcription, phenotypes and functions [17,18]. Here, we confirmed the pertinence of the respective parameters. Conditions for in vitro differentiation of all macrophage populations may be difficult to establish [9,10,25]. Yet, this and other studies have shown that distinct experimental conditions permit to induce the expression of surface determinants and cytokines/chemokines that were diagnostic for M1 and M2 macrophages. In fact, the induction of M1 or M2 macrophages was reflected by changes in macrophage-specific surface determinants (Table 1). With regard to functional parameters, differences in the secretion of, e.g., TNF-α and IL-1β by M1 and M2 macrophages were not spectacular, but marked for CXCl10, CCL5, and the (surface determinant) chemokine receptor CCR7. The transcription of cytokines and interleukins such as TNF-α, IL-1β, and IL-6 is regulated by the NF-κB pathway. Res and other anti-oxidants effected directly the expression of NF-κB factors ([40,51,52]; reviewed in [53]). Consequently, Res could regulate numerous prototype M1 macrophage genes (compared to M2 prototype genes). Yet, the activity of Res seems to be rather complex and cell-specific: Res enhanced the IL-6 expression in PBMCs, whereas it strongly impaired IL-6 expression in murine RAW264.7 macrophages [40,43,54]. This corroborates the overall anti-inflammatory effect of Res. We hypothesize that it might transiently increase secretion of pro-inflammatory cytokines in order to promote rapid resolution of the inflammatory response. It should be mentioned that Res increased CD206 surface deposition in M1-differentiated cells and therefore the shift to the M2 phenotype (Figure 4). This is in line with other studies where Res modulated CD206 expression [55] and acted on the M1/M2 macrophage balance at inflammatory conditions [56].

ω-3 PUFAs are membrane constituents and contribute to increased generation of EPA and DHA-derived resolvins but also prostaglandins (reviewed in [57]). Similar to Res, they further exemplify the dual role of nutrients as anti-inflammatory and pro-inflammatory agents [46]. DHA favoured M2 polarization and promoted the M1 to M2 macrophage switch [58,59].

Collectively, both Res and ω-3 PUFAs modulate the phenotype and the functions of M1 and M2 macrophages, which each substance having its particular and idiosyncratic activity profile. Importantly, when combined they two nutritional substances might have synergistic or antagonistic effects on macrophage phenotypes and functions. The in vivo significance of these results remains to be demonstrated by further investigations. It should be noticed that after absorption Res is rapidly metabolized and has only a short plasma half-life [47], yet the kinetics of ω-3 PUFAs indicate that they circulate in the plasma for weeks [48]. Res is supposed to have potent but transient and short-range effects on macrophage function, while ω-3 PUFAs have long-lasting effects. Both substances might have context-dependent but also macrophage population-specific effects [40]. We hypothesize that these distinct physiological profiles offer an advantage in targeting the effects of the two nutritional substances in transient (for Res) and prolonged or chronical (for omega-3 PUFAs) dietary supplementation.

## Figures and Tables

**Table 1:** Discriminating M1 and M2-specific macrophage populations by surface determinants and secreted interleukins/chemokines as reported in [2,9,17,21,25,26,27] and used in the present study to determine M1 and M2-lineage specific macrophage populations.

Surface Determinant	Macrophage Subtype
CD11b (integrin αM)	M2
CD11c (integrin αX)	M2
CD14 (LPS receptor)	M1
CD80 (B7-1)	M1
CD206 (macrophage mannose receptor)	M2
CD209 (DC-SIGN)	M2
CD274 (B7-H1)	M1
CCR2 (CC chemokine receptor 2)	M1
CCR7 (CC chemokine receptor 7)	M1
HLA-DR (MHC class II)	M2
Cytokines/Chemokines	Macrophage Subtype
IL-1β	M1
IL-6	M1
TNF-α	M1
IL-10	M2
IL-12p70	M1
CCL13/MCP4	M2
CCL18/PARC	M2
CCL20/MIP-3α	M2
CXCL10/IP-10	M1
CXCL1/GRO-α	M2

**Table 2:** Secretion of chemokines and cytokines by THP-1 cells, which were polarized by GM-CSF (to M1) and M-CSF (to M2), respectively, for 6 days and activated with LPS/INF-γ or IL-4/IL-13 for 24 h. Mean values ± standard deviation [pg/mL] of triplicates of a representative experiment (of three performed) are given. The M1/M2 ratio returns to the quotient of LPS/IFN-γ vs. IL-4/IL-13 induced secretion (assuming limit of detection of 1 pg/mL *).

Polarization	Activation	TNF-α	IL-1β	IL-6	IL-12p70	CCL13/MCP-4	CCL18/PARC	CCL20/MIP-3α	CXCL1/GROα	CXCL10/IP-10
6 days	24 h									
Medium	None	490 ± 20	91 ± 2	0.0 ± 0.0	0.0 ± 0.0	1.0 ± 0.0	4.0 ± 0.0	77,633 ± 7145	1.5 ± 0.3	0.0 ± 0.0
GM-CSF	None	1737 ± 203	374 ± 34	0.0 ± 0.0	0.0 ± 0.0	11 ± 1	5.0 ± 1.0	131,667 ± 10,017	10.7 ± 1.4	57 ± 7
M-CSF	None	628 ± 54	115±11	0.0 ± 0.0	0.0 ± 0.0	2.0 ± 0.0	3.0 ± 0.0	104,333 ± 4726	1.9 ± 0.2	0.0 ± 0.0
Medium	IL-4/IL13	252 ± 36	68 ± 2	0.0 ± 0.0	0.0 ± 0.0	304 ± 56	11 ± 1	61,933 ± 11,754	304.3 ± 56.2	61 ± 0
GM-CSF	IL-4/IL13	1111 ± 146	378 ± 66	0.0 ± 0.0	0.0 ± 0.0	331 ± 52	15 ± 3	105,000 ± 2000	331.3 ± 52.0	81 ± 10
M-CSF	IL-4/IL13	371 ± 24	98 ± 8	0.0 ± 0.0	0.0 ± 0.0	430 ± 14	11 ± 1	85,233 ± 5661	430.0 ± 13.7	66 ± 4
Medium	LPS/IFN-γ	25,533 ± 404	8140 ± 433	11,133 ± 551	9.0 ± 2.7	18 ± 1	15 ± 0	194,667 ± 11,846	17.6 ± 1.4	90,300 ± 15,455
GM-CSF	LPS/IFN-γ	33,567 ± 1872	11,800 ± 520	5730 ± 429	8.3 ± 1.4	24 ± 1	30 ± 3	279,000 ± 13,856	23.7 ± 1.0	81,500 ± 7892
M-CSF	LPS/IFN-γ	33,400 ± 781	9420 ± 243	11,467 ± 208	16.0 ± 9.3	20 ± 1	15 ± 1	236,333 ± 4163	19.9 ± 1.4	98,800 ± 15,029
M-CSF pol.	M1/M2 ratio	101	119	>5000 *	> 16	0.1	1.4	3.1	0.05	1480
GM-CSF pol.	M1/M2 ratio	30	31	>10,000 *	> 8	0.7	0.2	2.8	0.01	1006

**Table 3:** Effect of resveratrol and ω-3 PUFAs on chemokines and cytokine secretion by PMA-treated THP-1 cells, that were cultured for 48 h and, where indicated, activated with LPS/INF-γ or IL-4/IL-13 for 24 h before harvesting. Mean values ± standard deviation [pg/mL] of triplicates of a representative experiment (of three performed) are given. The M1/M2 ratio returns to the quotient of LPS/IFN-γ induced secretion *versus* IL-4/IL-13 induced secretion (assuming limit of detection of 1 pg/mL *).

Activation	Substances	TNF-α	IL-1β	IL-6	IL-12p70	CCL13/MCP-4	CCL18/PARC	CCL20/MIP-3α	CXCL1/GRO-α	CXCL10/IP-10
None	None	1443 ± 76	1410 ± 62	19.1 ± 1.9	27.9 ± 3.9	4.5 ± 0.4	20.9 ± 0.6	24,333 ± 737	5827 ± 339	197.7 ± 13.3
LPS/IFN-γ	None	22,100 ± 954	22,433 ± 1274	8740 ± 467	81.4 ± 16.3	218.5 ± 0.7	104.5 ± 2.1	38,867 ± 4654	33,400 ± 9617	>10,000
IL-4/IL-13	None	964 ± 150	5363 ± 3058	13.1 ± 12.6	125.5 ± 23.3	143.5 ± 0.7	27.6 ± 2.4	25,750 ± 6576	2510 ± 1994	230.0 ± 50.9
M1/M2 Ratio	None	23	4.2	667	0.6	1.5	3.7	1.5	13	>43 *
None	Res	718 ± 87	1247 ± 125	12.8 ± 1.1	115.5 ± 9.2	3.6 ± 0.6	24.1 ± 3.4	21,900 ± 2088	3297 ± 197	203.3 ± 9.5
None	PUFAs	268 ± 33	388 ± 67	2.9 ± 0.4	15.1 ± 2.4	2.6 ± 0.4	17.6 ± 1.2	6823 ± 1508	2697 ± 390	88.2 ± 6.5
Ratio untreat./Res		2.0	1.1	1.5	0.2	1.3	0.9	1.1	1.8	1.0
Ratio untreat./PUFA		5.4	3.6	6.3	1.9	1.7	1.2	3.6	2.2	2.2

**Table 4:** Secretion of chemokines and cytokines by PBMC cultured for 7 days in basal medium (M0), GM-CSF (M1 polarized) and M-CSF (M2-polarized). Mean values ± standard deviation [pg/mL] of triplicates of a representative experiment (of two performed) are given. The M1/M2 ratio returns to the quotient of LPS/IFN- γ induced secretion *versus* IL-4/IL-13 induced secretion. Secretion of chemokines and cytokines by PBMC cultured for 7 days in GM-CSF (M1-polarized) and M-CSF (M2-polarized). M1-polarized cells and M2-polarized cells were activated with LPS/IFN-γ and IL-4/IL-13, respectively, for 24 h. Data show the secreted metabolites during the 24 h activation period. Mean values ± standard deviation [pg/mL] of triplicates of a representative experiment (of three performed) are given. The M1/M2 ratio returns to the quotient of LPS/IFN- γ induced secretion *versus* IL-4/IL-13 induced secretion.

PBMC	TNF-α	IL-1β	IL-6	IL-10	IL-12p70	CXCL10/IP-10	CCL13/MCP-4	CCL18/PARC	CCL20/MIP-3α	CXCL1/GRO-α
M0	908 ± 38	2573 ± 337	28,133 ± 3066	5.6 ± 0.4	21.3 ± 3.4	126.3 ± 15.5	424 ± 158	16,966 ± 1464	490 ± 69	93,733 ± 16,815
M1-polarized	920 ± 13	1370 ± 140	25,433 ± 1357	4.6 ± 0.5	22.5 ± 5.2	65.2 ± 6.3	2023 ± 538	7563 ± 792	315 ± 39	90,000 ± 13,928
M2-polarized	886 ± 83	1550 ± 87	24,000 ± 1833	6.1 ± 0.6	27.1 ± 10.1	129.0 ± 4.0	1044 ± 115	12,000 ± 1135	455 ± 36	78,633 ± 8467
M1/M2 ratio	1.04	0.88	1.06	0.76	0.83	0.51	1.94	0.63	0.69	1.14
M1 polarized and activated	2065 ± 275	5045 ± 77	49,000 ± 9475	6.0 ± 1.4	19 ± 5	63 ± 32	272 ± 8	1915 ± 233	545 ± 172	30,450 ± 9828
M2 polarized and activated	1790 ± 141	5100 ± 735	50,600 ± 11,313	10.0 ± 2.2	6 ± 2	130 ± 30	908 ± 54	217 ± 4	525± 118	60,700 ± 14,283
M1/M2 ratio	1.15	1.0	1.0	0.6	3.0	0.5	0.3	8.8	1.0	0.5

## Data Availability

Not applicable.

## Supplementary Materials

The following supporting information can be downloaded at: https://www.mdpi.com/article/10.3390/biomedicines10071524/s1, Figure S1: Cytofluoremetric analysis of freshly isolated adherent PBMC for surface determinants.; Figure S2: Production of PGE_2_ by in vitro differentiated macrophages from PBMC; Table S1: Peak fluorescence intensity (PFI) data related to Figure 3; Table S2: Peak fluorescence intensity (PFI) data related to Figure 4.
