# Supplementary material for: Resveratrol and ω-3 PUFAs Promote Human Macrophage Differentiation and Function
Source: Biomedicines. 2022 Jun 28;10(7):1524. doi: 10.3390/biomedicines10071524 (PMC9313469; doi:10.3390/biomedicines10071524)
Supplement: Supplementary file 1 [file biomedicines-10-01524-s001.zip › biomedicines-1729940-supplementary.pdf]

## Supplementary Information

Figure S1. Cytofluoremetric analysis of freshly isolated adherent PBMC for surface determinants. Macrophages/ monocytes: red; lymphocytes: green; ‘intermediate cells (FCS/SSC between lymphocytes and macrophages/ monocytes): pink.

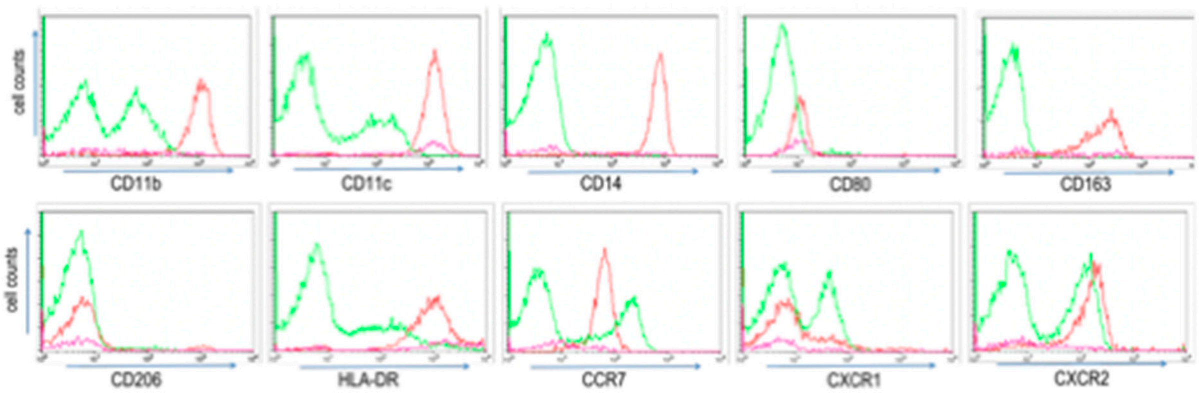


Figure S2. Production of $PGE_2$ by in vitro differentiated macrophages from PBMC.

Adherent cells isolated from peripheral blood were cultured in basal medium (M0 cells), GM-CSF (M1 cells) and M- CSF (M2) for M1 and M2 differentiation, respectively, for 7 days, followed by activation with LPS/IFN-γ or IL-4/ IL-13 for 24 h. Mean values ± standard deviation [pg/mL] of triplicates of a representative experiment (of four performed) are given. ** $p < 0.01$, * $p < 0.05$ (*versus* activated cells).

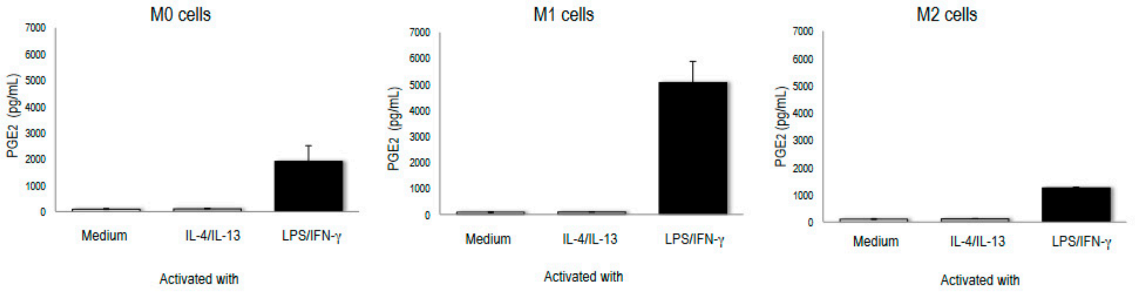

Table S1. Peak Fluoresecence Intensity (PFI) data related to Figure 3 of original article ***Resveratrol and omega-3 PUFAs Promote Human Macrophage Differentiation and Function*** °)

** p < 0.01, * p 0.01 < 0.05; NS = non-significant

| Surface Determinant | Macrophage population | Color-code in Figure 3 | PFI | -/+ st. dev. | M0 *vs* M1 | M0 *vs* M2 |
|---|---|---|---|---|---|---|
| CD11b | M0 | red | 320 | 52 | | |
| | M1 | green | 1280 | 234 | * | |
| | M2 | blue | 308 | 34 | | NS |
| CD14 | M0 | red | 292 | 121 | | |
| | M1 | green | 48 | 34 | * | |
| | M2 | blue | 409 | 122 | | NS |
| CD209 | M0 | red | 385 | 109 | | |
| | M1 | green | 250 | 23 | NS | |
| | M2 | blue | 6 | 4 | | * |
| CD274 | M0 | red | 112 | 23 | | |
| | M1 | green | 120 | 56 | NS | |
| | M2 | blue | 81 | 5 | | * |
| CCR7 | M0 | red | 108 | 12 | | |
| | M1 | green | 29 | 5 | ** | |
| | M2 | blue | 76 | 10 | | * |
| HLA-DR | M0 | red | 1998 | 201 | | |
| | M1 | green | 1090 | 12 | ** | |
| | M2 | blue | 1740 | 68 | | * |

Table S2. Peak Fluoerescence Intensity (PFI) data related to Figure 4 of original article ***Resveratrol and omega-3 PUFAs Promote Human Macrophage Differentiation and Function*** °)

** p < 0.01, * p 0.01 < 0.05; NS = non-significant

| | Surface Determinant | Treatment | Color-code in Figure 4 | PFI | -/+ st. dev. | none *vs* Resveratrol | none *vs* PUFAs |
|---|---|---|---|---|---|---|---|
| M1 | CD14 | none | black | 70 | 14 | | |
| | | resveratrol | red | 313 | 23 | ** | |
| | | omega-3 PUFAs | green | 4 | 1 | | ** |
| | CD206 | none | black | 1922 | 34 | | |
| | | resveratrol | red | 8590 | 245 | ** | |
| | | omega-3 PUFAs | green | 1267 | 234 | | NS |
| | CD274 | none | black | 100 | 12 | | |
| | | resveratrol | red | 258 | 120 | * | |
| | | omega-3 PUFAs | green | 69 | 41 | | NS |
| | HLA-DR | none | black | 523 | 56 | | |
| | | resveratrol | red | 1304 | 202 | * | |
| | | omega-3 PUFAs | green | 8967 | 299 | | ** |

| M2 | Surface Determinant | Treatment | Color-code in Figure 4 | PFI | -/+ st. dev. | | |
|---|---|---|---|---|---|---|---|
| | CD14 | none | black | 512 | 76 | | |
| | | resveratrol | red | 387 | 112 | NS | |
| | | omega-3 PUFAs | green | 221 | 34 | | * |
| | CD206 | none | black | 2041 | 38 | | |
| | | resveratrol | red | 348 | 152 | ** | |
| | | omega-3 PUFAs | green | 1756 | 76 | | NS |
| | CD274 | none | black | 107 | 12 | | |
| | | resveratrol | red | 246 | 19 | * | |
| | | omega-3 PUFAs | green | 84 | 54 | | NS |
| | HLA-DR | none | black | 536 | 82 | | |
| | | resveratrol | red | 8939 | 431 | ** | |
| | | omega-3 PUFAs | green | 2036 | 74 | | ** |

°) PFI refers to the fluorescence intensity at the peak of the curves indicated in Figure 3 and Figure 4
